# Effect of Visual Information on Active Touch During Mirror Visual Feedback

**DOI:** 10.3389/fnhum.2018.00424

**Published:** 2018-10-18

**Authors:** Narumi Katsuyama, Eriko Kikuchi-Tachi, Nobuo Usui, Hideyuki Yoshizawa, Aya Saito, Masato Taira

**Affiliations:** ^1^Department of Cognitive Neurobiology, Graduate School of Medical and Dental Sciences, Tokyo Medical and Dental University (TMDU), Tokyo, Japan; ^2^Center for Brain Integration Research (CBIR), Tokyo Medical and Dental University (TMDU), Tokyo, Japan

**Keywords:** mirror visual feedback, active touch, hardness perception, hand ownership, visual capture, multimodal integration, magnitude estimation

## Abstract

Several studies have demonstrated that observation of a dummy or mirror-reflected hand being stroked or moving at the same time as the hidden hand evokes a feeling that the dummy hand is one’s own, such as the rubber hand illusion (RHI) and mirror visual feedback (MVF). Under these conditions, participants also report sensing the tactile stimulation applied to the fake hands, suggesting that tactile perception is modulated by visual information during the RHI and MVF. Previous studies have utilized passive stimulation conditions; however, active touch is more common in real-world settings. Therefore, we investigated whether active touch is also modulated by visual information during an MVF scenario. Twenty-three participants (13 men and 10 women; mean age ± SD: 21.6 ± 2.0 years) were required to touch a polyurethane pad with both hands synchronously, and estimate the hardness of the pad while observing the mirror reflection. When participants observed the mirror reflection of the other hand pushing a softer or harder pad, perceived hardness estimates were significantly biased toward softer or harder, respectively, even though the physical hardness of the pad remained constant. Furthermore, perceived hardness exhibited a strong correlation with finger displacement of the mirrored, but not hidden, hand. The modulatory effects on perceived hardness diminished when participants touched the pad with both hands asynchronously or with their eyes closed. Moreover, participants experienced ownership of the mirrored hand when they touched the pad with both hands synchronously but not asynchronously. These results indicate that hardness estimates were modulated by observation of the mirrored hand during synchronous touch conditions. The present study demonstrates that, similar to passive touch, active touch is also modulated by visual input.

## Introduction

Previous studies have demonstrated that observation of a dummy hand being stroked or moving at the same time as the hidden hand evokes a feeling that the dummy hand is one’s own (rubber hand illusion, RHI; static dummy hand: Botvinick and Cohen, 1998; Ehrsson et al., 2004; moving dummy hand: Dummer et al., 2009; Kalckert and Ehrsson, 2012; Jenkinson and Preston, 2015). Similar illusory hand ownership is experienced when hand images are presented on video (Jeannerod, 2003; Tsakiris et al., 2006; Shimada et al., 2009), via mirror reflection (Bertamini et al., 2011; Medina et al., 2015), or using computer graphics (Hoermann et al., 2012; Bekrater-Bodmann et al., 2014; Kokkinara and Slater, 2014). Moreover, observing both dummy hands and fake hand images also elicits a change in the proprioceptive sensation of hand position (Botvinick and Cohen, 1998; Holmes et al., 2004, 2006; Tsakiris and Haggard, 2005). Additional studies have revealed that illusory ownership of the fake hand diminishes when the timing of tactile stimulation or motion is incongruent between the fake and hidden hands (e.g., Botvinick and Cohen, 1998; Kalckert and Ehrsson, 2012; Bekrater-Bodmann et al., 2014; Medina et al., 2015). These findings suggest that synchrony of the tactile stimulation or hand movement is crucial for the induction of illusory ownership.

In addition to perceiving the fake hand as one’s own, participants also experience the sensation that the hidden hand is receiving the same tactile stimulation as the fake hand. In the RHI, the dummy and hidden hands are stroked at the same time, allowing participants to perceive the tactile sensation at the stimulation site on the dummy hand, rather than on the hidden hand (Botvinick and Cohen, 1998; Pavani et al., 2000). After induction of the RHI, observation of the dummy hand being irradiated by a red laser (Durgin et al., 2007) or touched by an ice cube (Kanaya et al., 2012) elicits a warm or cool sensation, respectively, even though no thermal or neutral stimulation is delivered to the hidden hand.

Mirror visual feedback (MVF) refers to the phenomenon that the mirror reflection of a hand appears to be the hand hidden behind a mirror placed at the midline of the body. Many studies have indicated that sensitivity to tactile stimulation delivered to the hidden hand is modulated by observation of the mirror reflection of the other hand. For example, observing the mirror reflection of an intact hand impairs the detection threshold of vibratory stimulation delivered to the hidden hand (Harris et al., 2007). When participants move both hands back and forth at the same time and observe the mirror reflection of one hand, the perceived gap in the vibratory stimulation delivered to the hidden hand is exacerbated (Bultitude et al., 2016). Observation of an intact hand in the mirror reflection also alleviates pain in the hidden hand induced by laser or heat stimulation (Longo et al., 2009; Mancini et al., 2011). In contrast, observing the mirror reflection of a hand being stroked elicits an illusory tactile sensation on the corresponding site of the intact hidden hand (Ro et al., 2004; Takasugi et al., 2011; Hoermann et al., 2012). Furthermore, observation of the forearm being scratched attenuates an itchy sensation on the hidden hand induced via injection of histamine (Helmchen et al., 2013). As previously mentioned for the RHI, asynchronous (Async) tactile stimulation or hand movement abolishes illusory hand ownership and sensations associated with MVF (Durgin et al., 2007; Kanaya et al., 2012; Bultitude et al., 2016). Taken together, these findings indicate that tactile perception is mediated by vision.

In most previous studies regarding illusory sensations, tactile stimulation (e.g., stroking, vibratory, or thermal stimulation) was delivered passively while participants maintained their hand in a stable position on a desk. However, passive tactile stimulation is rarely experienced during daily life. Rather, we actively move our hands to obtain information regarding the shape, texture, hardness and viscosity of objects, among other properties. Tactile perception evoked by passive stimulation is referred to as passive touch, while the perception of object features obtained via exploratory hand movements is referred to as active touch. The finding that passive touch is mediated by vision indicates that active touch may also be modulated by visual information. Indeed, perceptions of shape, size and texture via active touch are improved by vision under certain conditions (Rock and Victor, 1964; Lederman and Abbott, 1981; Heller, 1982; Ernst and Banks, 2002; Whitaker et al., 2008). Moreover, previous studies have reported that illusory ownership of the dummy hand is stronger when participants observe the hand as moving rather than static (Dummer et al., 2009; Kalckert and Ehrsson, 2012).

Therefore, to determine whether active touch is mediated by visual information, we investigated whether the perception of hardness via active touch is affected by vision using an MVF experiment. For this purpose, we conducted two experiments. In Experiment 1, participants were required to touch a sponge pad with both hands in front of and behind a mirror placed in the parasagittal plane watching the mirror reflection, and estimate the hardness of a pad perceived by the hand behind the mirror. The modulation of visual information was analyzed by comparing the perceived hardness between conditions in which the seen and touched hardness of the pad was congruent and incongruent. In Experiment 2, we investigated whether participants can estimate the hardness of the pads based on visual cues only. Participants were presented with movies in which a hand is pushing a pad of different physical hardness and required to estimate the hardness without touching them. The visual cues in the movies utilized by participants for the hardness estimation were also investigated.

## Materials and Methods

We conducted two experiments in the present study. In Experiment 1 (*n* = 23), we examined the effect of vision on the perception of the hardness of polyurethane foam pads via active touch in an MVF scenario. In Experiment 2 (*n* = 13), we investigated whether participants could estimate the hardness of the pads based on visual cues only, as well as the types of cues utilized, when viewing movies of a hand pushing the pads.

This study was conducted in accordance with the World Medical Association’s Declaration of Helsinki. All procedures of this study were approved by the Ethical Committee for the Faculty of Dentistry of the Tokyo Medical and Dental University (TMDU; approval number: 681). All participants had normal or corrected-to-normal vision, were free of current or past neurological illness, and provided informed written consent prior to participation.

### Experiment 1

#### Participants

Twenty-three adults (13 men and 10 women; age: 21.6 ± 2.0 years; Edinburgh handedness score: 77.0 ± 36.5, mean ± SD) participated in Experiment 1.

#### Apparatus: Sponge Pad

**Figure 1:**
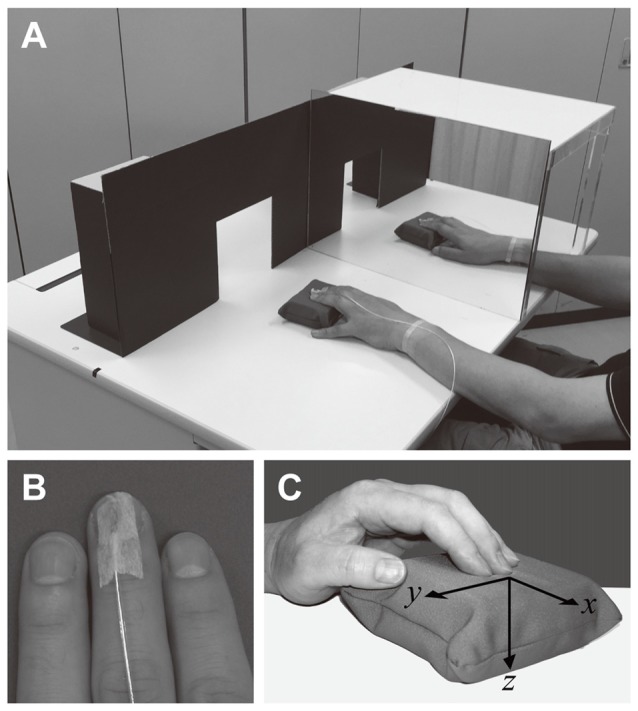
Panel **(A)** depicts the mirror visual feedback (MVF) scenario. Participants were required to watch the mirror so that they felt as if the hand in the mirror reflection was the hidden hand behind the mirror. An experimenter sitting at the opposite side of the desk (not shown) presented a foam pad to each hand of the participant through the rectangular window of the partition. Panel **(B)** shows the sensor of the electromagnetic tracker used to measure finger movements. The sensor was attached to the fingertip of the third finger of both hands using surgical tape. No tape was attached to the finger pad. The transmitter of the device was placed behind the partition such that it remained hidden from the participant. Panel **(C)** indicates the coordinates for the finger movements.

Three polyurethane foam pads (length × width × height: 9 × 7 × 4 cm) with different solidities were utilized (Figures 1A,C; Inoac Corporation, Nagoya, Japan). The pads were labeled in increasing order of hardness as P1, P2 and P3, respectively. The physical hardness of each pad was 1871.7, 3434.6 and 9803.9 N/m^2^, respectively. Each pad was wrapped in a green cloth, making it impossible for participants to judge the hardness of the pads based on appearance.

#### Apparatus: Mirror Box and Partition

A mirror (height × width: 30 × 50 cm) attached to an acrylic frame was placed in front of participants, such that the mirror aligned with the participants’ parasagittal plane (Figure 1A). The box was symmetrical, ensuring that either the left or right hand could be reflected by switching the direction of the mirror. A partition (30 cm × 90 cm) was placed on the desk, 50 cm in front of the participant (Figure 1A). There were two open windows (15 cm × 15 cm, 40 cm apart in center-to-center distance) on the partition through which an experimenter sitting on the opposite side of the desk could present each pad to the participant’s hands. The partition prevented the participant from seeing the next pad or the electromagnetic transmitter (see below) placed just behind it.

#### Behavioral Task: Monomanual Condition

The mirror box was not used in this condition. Each participant was instructed to place one hand on the desk (test hand) and the other under the desk. In each trial, the experimenter presented one of the three pads to the test hand through the open window of the partition. The participant was then required to place the central three fingers of the test hand on the top surface of the pad and retain the position for 3–4 s (pretrial period). Following the experimenter’s oral cue, the participant was required to press on the pad softly while watching his or her fingers in order to estimate the hardness of the pad. No instruction was given to participants about how to touch except a few who pushed the pad too strongly and quickly. Participants provided oral magnitude estimates of the perceived hardness (Gescheider, 1997). Any positive numbers, including decimals and fractions, could be used for the estimation. No time limit was enforced for the estimation. The answers were repeated orally by another experimenter in the room and recorded on an answer sheet. Each participant’s answers were also recorded using an electronic voice recorder (DS-50, Olympus Corporation, Tokyo, Japan) for offline verification.

#### Behavioral Task: Bimanual Condition

The mirror box was placed on the desk such that it aligned with the participant’s midline (Figure 1A). Participants were instructed to place one hand in front of and one hand behind the mirror, the latter of which remained invisible to the participant. They were also required to adjust the position of both hands so that the mirror reflection of a hand appears to be the hidden hand at the beginning of each trial. In a given trial, the experimenter presented only P2 to the hand at the back side of the mirror (test hand), while either P1, P2, or P3 was presented to the hand at the front side of the mirror (mirrored hand). After the pretrial period, participants were required to push the pad softly with the fingers of both hands, and to estimate the hardness of the pad perceived by the *test* hand. As in the single-hand condition, participants provided oral magnitude estimates of the perceived hardness in each trial. The answers were verified and recorded by the second experimenter and using a voice recorder. No time limit was enforced for the estimation.

##### Effect of Visual Input

**Figure 2:**
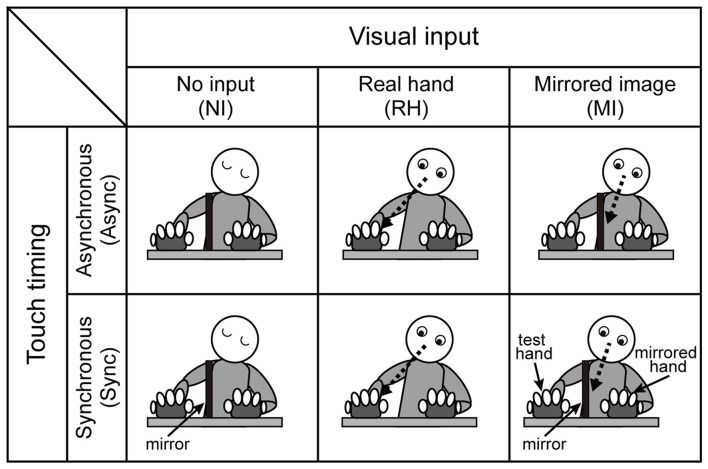
Schematic diagram of the behavioral task in the bimanual condition. Three visual (no input (NI), real hand (RH), mirrored image (MI)) and two timing (asynchronous (Async) and synchronous (Sync)) conditions were employed. The RH condition, which included both Async and Sync conditions, was blocked with the monomanual condition. The no-input and mirrored-image conditions were intermingled in the same block (see Table 1).

To control visual input, we employed the following three conditions (Figure 2): (1) no input (NI), in which participants were required to close their eyes while touching the pads; (2) real hand (RH), in which participants were asked to look at the fingers of the test hand while touching the pads (conditions similar to monomanual experiment without the mirror box); and (3) mirrored image (MI), in which participants were instructed to look at the reflection of the *mirrored* hand while touching the pads.

##### Effect of Touch Timing

We included two conditions to examine the effect of touch timing (Figure 2): (1) Async, in which participants were required to touch the pad in alternating fashion with each hand; and (2) Sync, in which participants were required to touch the pad at the same time with both hands. Thus, six (three visual × two timing) conditions were utilized in the bimanual experiment.

##### Effect of Handedness

To determine the effect of handedness on the perception of hardness, the test and mirrored hands were switched by changing the direction of the mirror box.

The seven conditions (one monomanual and six bimanual conditions) were presented in two blocks with (NI-Async, NI-Sync, MI-Async, MI-Sync) and without (monomanual, RH-Async, RH-Sync) the mirror box. Participants completed two blocks using each hand as the test hand, resulting in a total of four blocks for each participant (Table 1). In the block with the mirror box, the direction of the box was switched depending on the test hand. The order of the blocks was counterbalanced across participants. In each block, trials from each condition were executed three times in a pseudo-randomized order.

**Table 1:** Blocks in Experiment 1.

#	Test hand	Mirror box	Number of trials	Conditions
1	Left hand	not used	27	MONO, RH-Async, RH-Sync
2	Right hand	not used	27	MONO, RH-Async, RH-Sync
3	Left hand	used	36	NI-Async, NI-Sync, MI-Async,
				MI-Sync
4	Right hand	used	36	NI-Async, NI-Sync, MI-Async,
				MI-Sync

*Abbreviations: MONO, monomanual condition; NI, no visual input; MI, mirrored image; RH, real hand; Sync, synchronous; Async, asynchronous*.

#### Measurement of Finger Movements

Finger movements during the touch period were monitored using an electromagnetic tracker (3D Guidance trakSTAR, Ascension Technology Corporation, Shelburne, VT, USA). A small sensor (1 cm long) was attached to the nail plate of the third finger of both hands using surgical tape (Figure 1B). The conductive wire was fixed at the wrist using tape so as not to impede participants during the experiments. A transmitter yielding a static electromagnetic field was placed 50 cm in front of participants. The transmitter remained hidden behind the partition. The three-dimensional (3D) position in the *x*, *y* and *z* axes was measured at sampling rate of 80 Hz for each axis. The mean position for 3 s during the pretrial period was defined as the baseline in each trial. The positive values of the *x*, *y* and *z* coordinates were defined as follows: *x*, movement from participant toward the transmitter; *y*, movement from left to right; and *z*, movement from top to bottom (Figure 1C). Participants moved the fingers up and down several times during the touch period, and the peak and bottom of each stroke was determined using the *z* coordinate. The mean Cartesian distance between the peak and bottom of the strokes was calculated for each condition and designated as the overall measurement of finger movement during the touch period (i.e., displacement). We also calculated the number of strokes and duration of each stroke during the touch period. Finger movements could not be recorded for one participant due to improper operation of the devices. Therefore, finger movements were analyzed for 22 participants. Following the experiments, no participants reported being bothered or impeded by the touch sensors when estimating the hardness of each pad.

#### Data Analysis

The range of the perceived hardness scores reported using magnitude estimation varied between participants. Therefore, perceived hardness was normalized using the maximum value for each participant and averaged over all participants. In the monomanual condition (MONO), the perceived hardness was analyzed via a one-way repeated-measures analysis of variance (ANOVA) for the main effect of the pad (PAD, P1 vs. P2 vs. P3). In the bimanual condition, perceived hardness, displacement, number of strokes and stroke duration were analyzed using a three-way repeated-measures ANOVA for the main effects of visual input (VISUAL, NI vs. RH vs. MI), touch timing (TIMING, Async vs. Sync) and pad (PAD, P1 vs. P2 vs. P3) for each condition. *Post hoc* analyses were performed using Ryan’s method.

#### Hand Ownership Test

A questionnaire regarding hand ownership was administered after the blocks containing the MI condition. The following items were adopted from previous studies and translated into Japanese (Botvinick and Cohen, 1998; Longo et al., 2009):

1. It felt like I was looking directly at my hand rather than at a mirror image.
2. It felt like the hand I was looking at was my hand.
3. Did it seem like the hand you saw was a right hand or a left hand?
4. I felt like the position of my right hand changed.
5. I felt like the position of my left hand changed.
6. I felt as if the mirrored hand was someone else’s hand.
7. It seemed as if I might have more than one hand.

Participants were required to rate their agreement with each statement on a scale ranging from −3 (strongly disagree) to +3 (strongly agree). Ratings of 0 indicated that the participant neither agreed nor disagreed. For item 3, a different scale ranging from −100 (strongly left hand) to +100 (strongly right hand; 0: equally left and right hands) was used. Participants could respond with any intermediate value on a scale. Agreement or disagreement was tested by comparing the mean score with 0 using a one-sample *t*-test.

### Experiment 2

#### Participants

We performed power analysis to determine the sample size for this experiment by using G*Power ver. 3.1.9.2^1^ (Faul et al., 2007). We found that at least nine participants would be required to obtain the equivalent effect size to those of the MI-Sync condition in Experiment 1. As a result, 13 healthy participants (five men and eight women, mean age: 21.7 ± 0.8 years) who had not participated in Experiment 1 took part in Experiment 2. All participants were right-handed (Edinburgh handedness score: 91.2 ± 8.4).

#### Visual Stimulus

We prepared three movies depicting a right hand pushing the P1, P2 and P3 pads, respectively. An experimenter sat at a desk and pushed each of the pads using the right hand, which was placed on the desk. The desk and pads were the same as those used in Experiment 1. The experimenter pushed the pads in pace with a beep delivered at 0.8 Hz, the mean frequency of the finger strokes under the Sync conditions in Experiment 1. The finger movements were monitored using an electromagnetic tracker and the finger displacement was adjusted so that it matched the mean displacement of the mirrored hand for the pads under the Sync condition of Experiment 1 (9.4, 6.4 and 4.1 mm for P1, P2 and P3, respectively). Sound-free recordings were obtained using a digital video camera (HDR-XR 520V, Sony, Tokyo, Japan). The composition of the movies was adjusted so that the hand in the movies would appear to be that in the mirror reflection during Experiment 1 (Figures 1A, 8A). The spatial and temporal resolution of the movies were 440 × 550 pixels (height × width) and 50 fps, respectively. The sensor of the electromagnetic tracker attached to the third finger and the wire were also present in the movies.

**Figure 3:**
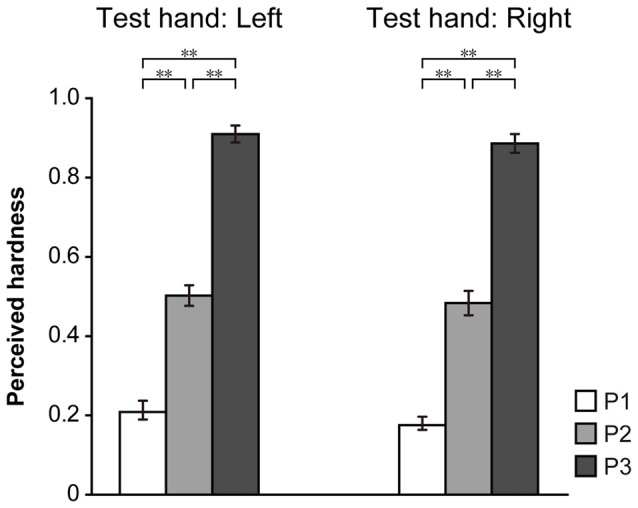
Results for the monomanual condition. Hardness estimates, obtained using the left and right hand, are shown. The perceived hardness was normalized by the maximum value of each participant and averaged across all participants. Error bar: standard error (SE). **p < 0.01.

**Figure 4:**
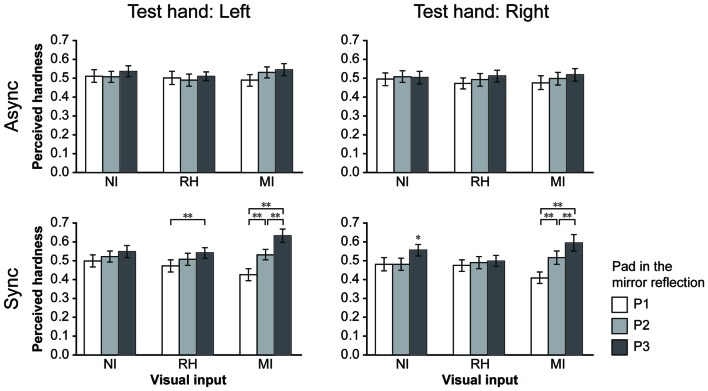
Results for the bimanual condition. The perceived hardness was normalized by the maximum value of each participant and averaged across all participants. Mean perceived hardness values were analyzed via repeated-measures analysis of variance (ANOVA) for the main effects of visual input (NI vs. RH vs. MI), touch timing (Async vs. Sync) and pad (P1 vs. P2 vs. P3) for each hand. NI, no input; RH, real hand; MI, mirrored image; Async, asynchronous; Sync, synchronous. Error bar: SE; **p < 0.01, * *p* < 0.05.

**Figure 5:**
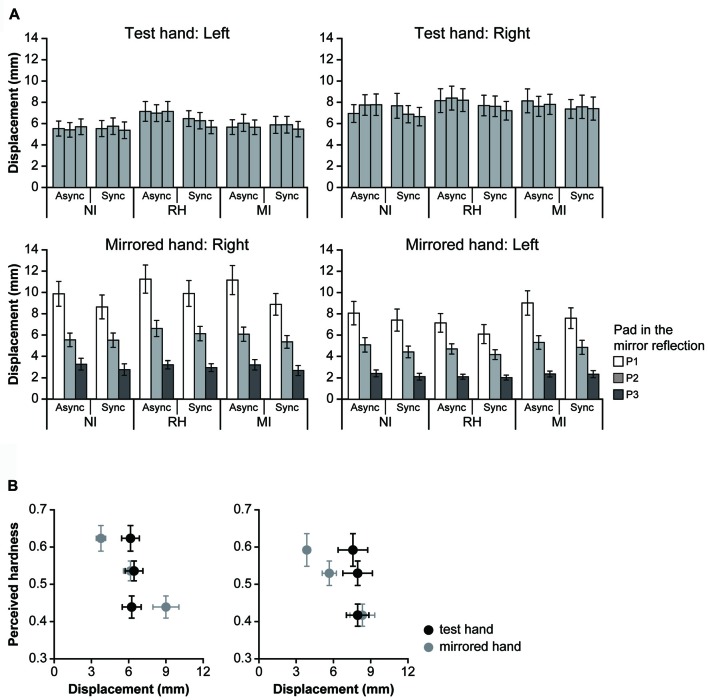
Finger movements during the touch period. Panel **(A)** shows the mean finger displacement in the *z* axis (vertical direction) of the test (upper row) and mirrored hands (lower row). Panel **(B)** shows the relationship between perceived hardness and displacement of the test and mirrored hands under the MI-Sync condition. The perceived hardness values are the same as those shown in **(A)**. NI, no input; RH, real hand; MI, mirrored image; Async, asynchronous; Sync, synchronous. Error bar: SE.

**Figure 6:**
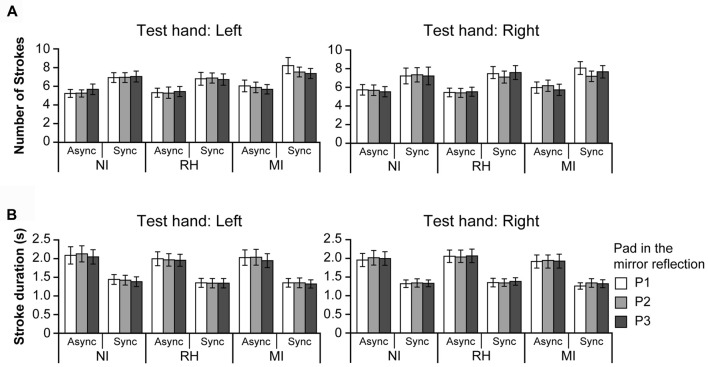
Panel **(A)** shows the number of strokes made by the test hand during the touch period. Panel **(B)** shows the stroke duration for the test hand. There was no difference in the number of strokes or stroke duration between the test and mirrored hands; therefore data are presented for the test hand only. NI, no input; RH, real hand; MI, mirrored image; Async, asynchronous; Sync, synchronous. Error bar: SE.

**Figure 7:**
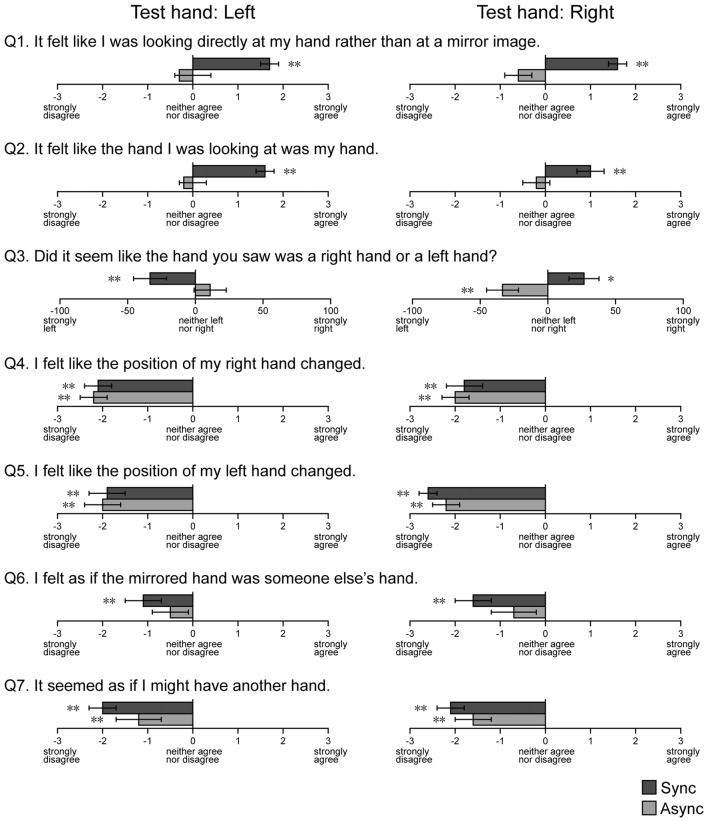
Results of the hand ownership test. Questions regarding hand ownership were presented after blocks containing the MI conditions. Async: asynchronous, Sync: synchronous. Error bar: SE. **p < 0.01, *p < 0.05.

**Figure 8:**
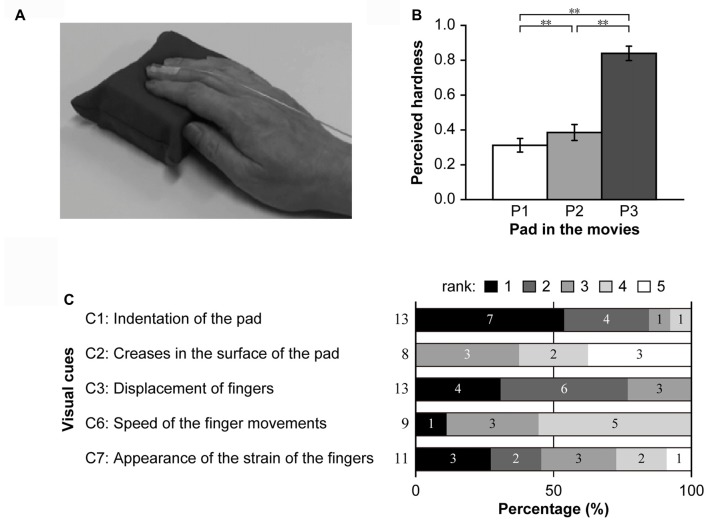
Panel **(A)** includes a representative screenshot from a movie presented in Experiment 2. Panel **(B)** shows the results of the hardness estimation test. The means of the estimated hardness values of P1, P2 and P3 over all participants are shown. **p < 0.01. Panel **(C)** illustrates the visual cues utilized by at least two participants in the hardness estimation test. The percentages of the rank orders for each cue are shown on a scale ranging from black (first) to white (fifth). Numbers at the left side and inside the bar graphs indicate the total number of participants who checked the cue and the number of participants who ranked the cue in the order indicated, respectively.

#### Hardness Estimation

Participants were asked to sit at a desk with their chins fixed to a chin rest. A 19-inch liquid crystal display (Flex Scan L767, EIZO Corporation, Ishikawa, Japan) was placed on the desk, 60 cm in front of the participant. Participants initiated the movie by hitting the space bar on a keyboard with the right hand. One of the three movies in which the P1, P2, or P3 was pushed by a hand was presented in each trial. Participants were required to watch the movie and estimate the hardness of the pad observed. Participants were allowed to hit the space bar at any time during the movie and orally report magnitude estimates of the pad’s hardness, as in Experiment 1. Each movie was presented seven times in pseudo-randomized order. The perceived hardness was normalized by the maximum value for each participant and analyzed via one-way repeated-measures ANOVA for the main effect of PAD (P1 vs. P2 vs. P3).

After the hardness estimation experiment, we assessed the visual cues utilized by each participant using the following 10 items (C1–C10):

C1. Indentation of the pad.
C2. Creases in the surface of the pad.
C3. Displacement of fingers into the pad.
C4. Creases in the fingers.
C5. Changes in the color of fingers.
C6. Speed of the finger movements.
C7. Appearance of the strain of the fingers.
C8. Motions of the wire from the sensor.
C9. Motions of the shadows cast on the floor.
C10. Other cues.

Participants provided open-ended responses to C10 regarding any cues other than those described in C1–C9. Participants were asked to check all cues that applied and rank them in order of relevance to their estimates. Cues checked by at least two participants were selected, following which their rankings were analyzed.

### Statistical Analysis

In the present study, all statistical analyses were performed at significance level of *p* < 0.05.

## Results

### Experiment 1

#### Monomanual Condition

The results of the monomanual condition are shown in Figure 3. The ordinate indicates the mean of the perceived hardness normalized to the maximum in each participant. A one-way repeated-measures ANOVA indicated a significant main effect of PAD (P1 vs. P2 vs. P3) in both the left (*F*_(2,42)_ = 82.1, *p* < 0.001) and right (*F*_(2,42)_ = 100.8, *p* < 0.001) hands. A *post hoc* Ryan’s test indicated that the difference between P1 and P2, P1 and P3 and P2 and P3 was significant in both hands (*p* < 0.001 for all comparisons). These results indicate that participants were able to discriminate the hardness of the three pads based on visual and haptic information.

#### Bimanual Conditions

In the bimanual conditions, participants were required to provide hardness estimates (test hand) after touching the pad with both hands under different visual and timing conditions. They touched the P2 (medium hardness) only with the test hand in all trials. The perceived hardness averaged across participants is shown in Figure 4. A repeated-measures ANOVA was performed for the main effect of VISUAL (NI vs. RH vs. MI), TIMING (Async vs. Sync) and PAD conditions (P1 vs. P2 vs. P3). When the left hand was used as the test hand (left column), we observed a significant main effect of PAD (*F*_(2,44)_ = 7.84, *p* < 0.005), and a significant interaction between the VISUAL and PAD conditions (*F*_(4,88)_ = 6.82, *p* < 0.001) TIMING and PAD conditions (*F*_(2,44)_ = 12.96, *p* < 0.001), and among all conditions (*F*_(4,88)_ = 4.68, *p* < 0.005). *Post hoc* analysis revealed that perceived hardness was significantly modulated under the MI-Sync and RH-Sync conditions (bottom), but not under other conditions. Under the MI-Sync condition, there was a significant difference in perceived hardness between P1 and P2, P1 and P3 and P2 and P3 (*p* < 0.001 for all comparisons). These findings indicate that participants perceived P2 as harder or softer with the left hand when they observed a mirror reflection of the right hand pushing P3 or P1 at the same time. Under the RH-Sync condition, the perceived hardness was significantly greater for P3 than for P1 (*p* < 0.005). Similarly, when the right hand was used as the test hand (right column), an ANOVA revealed a significant main effect of PAD (*F*_(2,44)_ = 9.71, *p* < 0.001), and a significant interaction between the VISUAL and PAD conditions (*F*_(2,44)_ = 9.71, *p* < 0.001), TIMING and PAD conditions (*F*_(2,44)_ = 10.68, *p* < 0.001) and among all three conditions (*F*_(4,88)_ = 6.03, *p* < 0.001). *Post hoc* analysis revealed that perceived hardness differed significantly under the MI-Sync and NI-Sync conditions (bottom). Under the MI-Sync condition, there was a significant difference in perceived hardness between P1 and P2, P1 and P3 and P2 and P3 (*p* < 0.001 for all comparisons). Under the NI-Sync condition, the perceived hardness of P3 was significantly greater than P1 (*p* < 0.01) and P2 (*p* < 0.001). Thus, significant visual modulation of perceived hardness was observed under the MI-Sync condition for both hands. To quantify the extent of visual modulation, we calculated the modulation index (MI) for the perceived hardness of P1 and P3 using the following equation:

$$\text{Modulation index}\left({\text{MI}}_{i}\right)=\frac{\left|S_{i}-S_{2}\right|}{S_{2}},$$

where *S*_i_ indicates the perceived hardness when the pad in the mirror reflection is P*i* (*i* = 1 or 3). Thus, the MI indicates the percent change of the perceived hardness when participants observed P1 or P3 pushed in the mirror reflection compared with when they observed P2. The mean MI_1_ and MI_3_ values across all participants were 0.24 ± 0.17 and 0.27 ± 0.28 (mean ± SD) for the left hand, and 0.21 ± 0.16 and 0.30 ± 0.38 for the right hand, respectively. Welch’s *t*-test revealed no significant difference between the MI_1_ and MI_3_ for either hand (left: *t*_(36.3)_ = 0.56, *p* = 0.58; right: *t*_(29.1)_ = 0.98, *p* = 0.34), suggesting that the level of visual modulation was nearly identical when participants perceived P2 as softer or harder.

#### Finger Movements During Touch

To determine whether the modulation of perceived hardness was caused by visual information rather than finger movement during the touch period, we investigated the relationship between perceived hardness and finger movement (Figure 5A). The displacement was calculated for both hands when the left or right hand was used as the test hand. When the left hand was used as the test hand (left column), a three-way ANOVA revealed no main effects on the displacement of the test hand (top left); however, a significant interaction was observed between the VISUAL and TIMING conditions (*F*_(2,42)_ = 6.37, *p* < 0.01). Analysis of the simple main effect for the interaction indicated that the displacement was significantly greater under the RH-Async than under the RH-Sync condition (*F*_(1,63)_ = 10.64, *p* < 0.01). *Post hoc* analysis also indicated that displacement in the RH condition was significantly greater than the NI and MI conditions under the Async conditions (*p* < 0.01 for both comparisons), and that there was no significant difference in that between the NI and MI conditions (*p* = 0.61). No difference in displacement was observed under the Sync conditions. However, an ANOVA revealed significant main effects of TIMING (*F*_(1,21)_ = 10.65, *p* < 0.01) and PAD (*F*_(2,42)_ = 55.39, *p* < 0.0001), and an interaction between the two (*F*_(2,42)_ = 5.42, *p* < 0.01), with regard to the displacement of the mirrored hand (bottom left). *Post hoc* analysis revealed significant differences in displacement between P1 and P2, P1 and P3 and P2 and P3 (*p* < 0.001 for all comparisons). Significant differences in displacement between the NI and RH, RH and MI and MI and NI conditions were also observed for the Async and Sync conditions (*p* < 0.001 for all comparisons). When the right hand was used as the test hand (right column), ANOVA revealed a significant main effect of TIMING (*F*_(1,21)_ = 5.56, *p* < 0.03), and an interaction between TIMING and PAD (*F*_(2,42)_ = 4.87, *p* < 0.02), for the test hand (top right). Analysis of the simple main effect for the interaction revealed that displacement during the Async condition was significantly greater than that during the Sync condition for both P2 (*F*_(1,63)_ = 4.95, *p* < 0.03) and P3 (*F*_(1,63)_ = 10.81, *p* < 0.01). *Post hoc* analysis indicated that the displacement of P1 was significantly greater than that of P3 under the Async condition (*p* < 0.01). We observed significant main effects of TIMING and PAD (*F*_(2,42)_ = 42.65, *p* < 0.0001) on the displacement of the mirrored hand (*F*_(1,21)_ = 6.71, *p* < 0.02), and an interaction between the two (*F*_(2,42)_ = 3.26, *p* < 0.05). We also observed a significant interaction between the VISUAL and PAD conditions (*F*_(4,84)_ = 2.48, *p* < 0.0; bottom right). *Post hoc* analysis revealed a significant difference between P1 and P2, P2 and P3 and P3 and P1 under the NI, RH and MI conditions (*p* < 0.001 for all comparisons). Significant differences in displacement were also observed between P1 and P2, P2 and P3 and P3 and P1 under the Async and Sync conditions (*p* < 0.001 for all comparisons).

Next, we examined the relationship between perceived hardness and displacement of the test and mirrored hands under the MI-Sync condition (Figure 5B). When the left hand was used as the test hand (left), perceived hardness decreased as the displacement of the mirrored hand increased, whereas that of the test hand was constant. This was also observed when the right hand was used as the test hand (right). These results indicate that perceived hardness under the MI-Sync conditions strongly depends on displacement of the mirrored hand, but not of the test hand.

#### Number of Strokes and Stroke Duration During Hardness Estimation

We also investigated the number of strokes required for participants to provide hardness estimates (Figure 6A). Because there was no difference in the number of strokes between the test and mirrored hands, data are presented for the test hand only. When the left hand was used as the test hand (left), ANOVA revealed a significant main effect of TIMING (*F*_(1,21)_ = 26.48, *p* < 0.001), and an interaction between the VISUAL and PAD conditions (*F*_(4,84)_ = 2.48, *p* < 0.05). The number of strokes was significantly greater under the Sync condition (6.64) than Async condition (5.59). *Post hoc* analysis revealed that the number of strokes for P1 was significantly greater under the MI condition than under the NI and RH conditions (*p* < 0.01 for both comparisons). In addition, the number of strokes under the MI condition was significantly greater than that under the NI condition (*p* < 0.01).

When the right hand was used as the test hand (right), ANOVA revealed a significant main effect only for the TIMING condition (*F*_(1,21)_ = 22.40, *p* < 0.001). The number of strokes was significantly greater under the Sync condition (6.90) than under the Async condition (5.64), although no interactions were observed. These results indicate that more strokes were required for participants to estimate the hardness of the pads during the Sync conditions than the Async conditions.

We further analyzed the stroke duration required for hardness estimates. Because there was no difference in mean stroke duration between the test and mirrored hands, data are presented for the test hand only. When the left hand was used as the test hand (Figure 6B, left), an ANOVA revealed a significant main effect of TIMING (*F*_(1,21)_ = 30.5, *p* < 0.001). The mean stroke duration was significantly longer under the Async condition (2.02 s) than under the Sync condition (1.36 s). ANOVA also revealed a significant main effect of PAD (*F*_(2,42)_ = 3.64, *p* < 0.05), although *post hoc* analysis revealed no significant differences in mean stroke duration.

When the right hand was used as the test hand (Figure 6B, right), ANOVA revealed a significant main effect of TIMING only (*F*_(1,21)_ = 34.97, *p* < 0.001). Stroke duration was significantly longer under the Async condition (1.99 s) than the Sync condition (1.33 s), irrespective of visual input. Taken together, these results indicate that participants pushed the pad faster and for a shorter duration under the Sync conditions than the Async conditions.

#### Hand Ownership Test

We investigated the sense of hand ownership under the MI conditions using a questionnaire (Figure 7). For the first question (Q1), which asked participants to report the condition of the visual image observed during the touch period, participants tended to report that they felt like they were looking at their own hand rather than a mirror reflection during the Sync condition, when both the left (*t*_(22)_ = 7.80, *p* < 0.001) and right (*t*_(22)_ = 6.75, *p* < 0.001) hands were used as the test hand. However, no such findings were observed for the Async condition (*t*_(22)_ = 0.72, *p* = 0.24 for left hand, *t*_(22)_ = 1.70, *p* = 0.052 for right hand). For the second question (Q2), which asked participants to report the condition of the hand observed in the mirror reflection, participants tended to report that they felt like they were looking at their own hand during the Sync condition, when both the left (*t*_(22)_ = 10.56, *p* < 0.001) and right (*t*_(22)_ = 3.56, *p* < 0.001) hands were used as the test hand. No results were observed for the Async condition (*t*_(22)_ = 0.74, *p* = 0.24 for left hand, *t*_(22)_ = −0.58, *p* = 0.28 for right hand). For the third question (Q3), participants reported that they felt as if they were looking at their own left and right hands when they observed mirror images (right: *t*_(22)_ = 2.68, *p* < 0.01; left: *t*_(22)_ = 2.36, *p* < 0.05). Next, we examined whether responses to Q1 and Q2 were correlated with the level of visual modulation under the MI-Sync and MI-Async conditions. However, no correlations were observed under either condition. Participants did not provide uniform responses to other questions (Q4–7) for either the Sync or Async condition. Only the answer to Q6 under the Async condition was non-significant for both hands, and all other answers were significant at *p* < 0.01. This result indicates that participants felt like the hand in the mirror reflection was their hidden hand under the MI-Sync condition but not the MI-Async condition.

### Experiment 2

Experiment 1 showed that when participants observe the mirror reflection of a hand pushing a soft or hard pad, the hardness of the pad perceived by the hidden hand is significantly biased toward soft or hard, respectively. The result suggested that perceived hardness by active touch is modulated by visual information; however, this interpretation presupposes that participants can estimate the hardness of the pad under a condition in which vision is the only available information. Moreover, it remains to be elucidated what kind of visual cues participants utilized for the hardness estimation. To investigate these questions, we conducted Experiment 2.

In this experiment, participants were required to observe movies in which a pad is pushed by a right hand and estimate the hardness of the pad based on visual cues only (Figure 8A). The perceived hardness of the pads in the movies is shown in Figure 8B. One-way ANOVA revealed a significant main effect of PAD (*F*_(2,12)_ = 252.50, *p* < 0.001). *Post hoc* analysis using Ryan’s test revealed a significant difference in perceived hardness between P1 and P2, P1 and P3 and P2 and P3 (*p* < 0.01 for all comparisons). This result indicated that participants could discriminate the hardness of the pads in the movies based on visual cues only.

Visual cues utilized for hardness estimates from at least two participants were assessed using a questionnaire. Among the 10 cues listed, no participants checked C8–10. C4 and C5 were checked by one participant each. Thus, subsequent analyses focused on C1, C2, C3, C6 and C7. Figure 8C shows the ratio of participant rankings for each cue. C1 and C3 were checked by all participants, and >75% of participants ranked these cues as the most and second-most utilized visual cues. C7 was ranked as the primary or secondary cue by half of the participants who selected the cue. Most participants ranked C2 and C6 as less important. These results indicate that participants utilized visual cues regarding both the pad and fingers when estimate hardness based on movie clips, especially indentation in the pad and finger movements/strain.

## Discussion

### Visual Information Modulates Perceived Hardness During Active Touch

In the present study, we investigated whether the perception of hardness during active touch is affected by visual input using an MVF scenario. In Experiment 1, participants touched a polyurethane foam pad with the hidden hand (test hand) while watching a mirror reflection of the other hand (mirrored hand) push pads of different solidities (MI condition). Following this, they provided estimates regarding the hardness of the pad touched by the test hand. When they touched the pad with both hands at the same time (MI-Sync condition), participants reported perceiving a softer or harder pad, even though the test hand was subjected to P2 in all trials. The perceived hardness exhibited a correlation with finger displacement of the mirrored hand, but not with that of the test hand. This modulatory effect disappeared when participants touched the pad with both hands at different times (MI-Async condition), and when participants touched the pad while looking at their own hand (RH conditions) and with their eyes closed (NI conditions), regardless of touch timing. These results indicate that observing one’s own hands touch pads of different solidities in a synchronous manner induced visual modulation of the perceived solidity of the pad. In addition, our results indicated that participants experienced illusory ownership of the mirrored hand under the MI-Sync condition but not under the MI-Async condition. The illusory hand ownership toward mirror reflection has been reported in previous studies. Illusory ownership is induced by watching a mirror placed in the parasagittal plane at the midline (Longo et al., 2009; Mancini et al., 2011; Takasugi et al., 2011; Hoermann et al., 2012; Jenkinson and Preston, 2015) or in the fronto-parallel plane at a distance (Bertamini et al., 2011). Taken together, the present results demonstrate that participants felt as if they touched the same pad with the hidden and mirrored hands. In accordance with previous findings regarding passive touch (Ehrsson et al., 2004; Durgin et al., 2007; Longo et al., 2008, 2009, 2012; Bertamini et al., 2011; Mancini et al., 2011; Takasugi et al., 2011; Hoermann et al., 2012; Kanaya et al., 2012; Bekrater-Bodmann et al., 2014; Medina et al., 2015), these findings suggest that active touch was modulated by visual input. Furthermore, it has been shown that illusory ownership can be induced not only in hands, but also in lower limbs (MaCabe et al., 2005) and in full-body by watching the participant’s own body via MVF (Preston et al., 2015). These results suggest that the illusory sensation by active touch may be extended to the other limb and full-body.

Using virtual reality techniques, previous studies have investigated the effect of vision on the perception of solidity (Kuschel et al., 2010; Cellini et al., 2013). In these studies, visual information was presented using a computer monitor, on which a deformable surface was pushed by non-corporeal objects (cylinder and sphere) rather than a hand. Haptic information was provided by squeezing two levers with simulated repulsive force (Kuschel et al., 2010) or touching silicone models with the hand (Cellini et al., 2013). The authors reported that perceived solidity was biased towards harder or softer when the indentation of the virtual surface was smaller or larger, respectively, consistent with the findings of the present study. However, these studies did not investigate whether participants experienced ownership of the cylinder or sphere pressing on the surface. Early studies indicated that biologically plausible characteristics of the dummy hand—such as the position, angle and color—are important for the induction of illusory ownership in the RHI (Pavani et al., 2000; Tsakiris and Haggard, 2005; Tsakiris, 2010; Ide, 2013). However, recent studies have revealed that hand ownership can be experienced when viewing computerized images of non-biological objects when the motion of the objects and hidden hand is synchronized (Short and Ward, 2009; Ma and Hommel, 2015). Therefore, participants may have experienced “hand ownership” of the cylinder and ball in these previous studies.

In Experiment 2, we examined whether participants could estimate the hardness of the pad based on visual cues only. When participants observed movies in which a right hand pushed one of the three pads, they were able to correctly discriminate the hardness of the pads. These findings are consistent with those of a previous study in which participants provided reliable magnitude estimates of the compliance of a rubber spaceman when directly observing the exploratory movements of another person (Drewing et al., 2009). Moreover, our results indicated that participants used visual cues regarding both the pad (indentations and creases on the surface) and fingers (displacement and strain) to obtain hardness estimates. Previous studies have suggested that indentations of the deformable surface represent a minimal visual cue for the perception of hardness (Kuschel et al., 2010; Cellini et al., 2013). Our findings suggested that participants utilize visual cues other than indentation when they are available. Thus, further studies are required to determine the contribution of different visual cues to the perception of hardness.

### Possible Cortical Mechanisms

Previous studies have proposed the cortical mechanisms underlying the illusory hand ownership under the RHI. Visual and tactile information about the dummy hand and participant’s hidden hand receiving a synchronous tactile stimulation is first processed in the unimodal sensory areas, and then integrated in the multimodal region of the posterior parietal cortex (PPC; Ehrsson et al., 2004; Pasalar et al., 2010; Gentile et al., 2011). There is a discrepancy between the seen and felt position of the stimulation; however, the brain attempts to resolve this discrepancy based on visual input, and determines that the dummy hand receiving the tactile stimulation is one’s own hand (Makin et al., 2008; Serino and Haggard, 2010; Tsakiris, 2010). Visual dominance in multimodal integration has been shown in many previous studies. The following examples show that illusory hand ownership can be induced even when somatic sensation inputs from the periphery are lost by amputation (Ramachandran and Rogers-Ramachandran, 1996) and cervical spinal cord injury (Lenggenhager et al., 2013; Pazzaglia et al., 2016). After the integration of the visual and tactile information, illusory hand ownership is represented in the brain. Evidence has revealed that the PPC and the premotor cortex (PMC) may be involved in the representation of illusory hand ownership (Ehrsson et al., 2004, 2005; Bekrater-Bodmann et al., 2014). Consequently, participants experience the tactile sensation on the stimulation site of the dummy hand. It has been suggested that the primary (SI) and secondary (SII) sensory areas may be involved in the perceived tactile sensation, and this may be reinforced by an interaction between the sensory areas and PPC and PMC that represent illusory hand ownership (Makin et al., 2008; Serino and Haggard, 2010; Tsakiris, 2010).

In active touch under the MVF (for example, the MI-Sync condition in the present study), participants move both hands synchronously, and the proprioceptive information is involved in the multimodal integration process in the PPC. The mirror reflection moving with the same timing as the hidden hand is determined as one’s own hand; therefore, participants felt as if they had touched the pad that is being touched in the mirror reflection by the hidden hand. As described earlier, the biologically plausible characteristics of the dummy hand is important for the induction of the illusory ownership in the RHI. In this context, the MVF may be advantageous, because the hand image in the mirror reflection is one’s own hand (although it is the opposite hand), and it is easy to perform synchronized movements with the mirrored and hidden hands.

In the present study, the fingers of the test and mirrored hand moved in the opposite direction and no illusory ownership nor the visual modulation of the perceived hardness was observed under the MI-Async condition. There were discrepancies in the displacement of the finger movements between the test and mirrored hands under the MI-Sync condition when participants touched P1 and P3 with the mirrored hand. The results of the Experiment 2 indicated that participants realized the discrepancy and utilized it for the hardness estimation. Nevertheless, the illusory ownership and the visual modulation of the perceived hardness were induced under this condition. If vision is the dominant information source for multimodal integration, one may argue that these results are contradictory. One possible explanation for this discrepancy is that finger movements in the same direction may be more biologically plausible than those in a different direction. When a fake hand moves in the same direction with the same timing as the hidden hand, it may be considered to belong to one’s body irrespective of some discrepancies in displacement and speed. On the contrary, when a fake hand shows different movements compared with hidden hands, the discrepancy in visual and proprioceptive information between both hands cannot be negligible. This may prevent the embodiment of the fake hand. Compared with the RHI using a static dummy hand, little is known about the detailed conditions for the induction of illusory hand ownership toward the moving dummy hand (Dummer et al., 2009; Kalckert and Ehrsson, 2012; Jenkinson and Preston, 2015). This may be a problem for future experiments in the field.

Finally, our preliminary result suggests that the SI and SII may be involved in the hardness perception by active touch under the MVF (Kim et al., 2018) as well as in the RHI. There are considerable differences in the cortical processes associated with active and passive touch (Gibson, 1962). For example, motor commands and proprioception are associated with active but not passive touch. Moreover, the effects of attention differ between active and passive touch. Results from the present study suggest that, despite these differences, the illusory hand ownership and visual modulation during active and passive touch may share similar neuronal mechanisms.

### Effect of Bimanual Coordination

When we move our hands in a different way, the movements of one hand can affect the motion of the other hand. For example, when the left and right hands are moved in linear and circular trajectories, respectively, the trajectory drawn by the left hand becomes more circular, while that drawn by the right hand gradually becomes more linear. This phenomenon is referred to as bimanual coordination (Franz, 1997; Swinnen and Wenderoth, 2004).

In the present study, participants touched pads of different solidities with both hands. The amplitude of finger displacement during touch largely depends on the physical hardness of the pads, such that softer pads result in greater amplitude of finger displacement. When participants touch a pad with one hand, the pressure applied to the finger pads increases as the finger displacement increases, allowing the participants to perceive the pad as harder. Therefore, one may argue that the modulation of the perceived hardness observed under the MI-Sync condition was caused by changes in the finger movements of the test hand induced by bimanual coordination rather than visual information. However, this is not plausible for several reasons. First, modulation of the perceived hardness was prominent only under the MI-Sync condition, whereas the effect of bimanual coordination would be expected under the NI-Sync and RH-Sync conditions. Second, there was no difference in the finger displacement of the test hand even when the mirrored hand pushed the harder and softer pads under the MI-Sync condition, suggesting that the effect of bimanual coordination was negligible. Finally, modulation of perceived hardness observed under the MI-Sync condition differed from that predicted by bimanual coordination. As previously mentioned, the motions of both hands become more similar due to bimanual coordination, suggesting that touching a softer pad with the mirrored hand would result in increased finger displacement of the test hand, allowing the pad to be perceived as harder. However, the perceived hardness was significantly smaller when participants pushed the softer pad with the mirrored hand. Similarly, when participants pushed the harder pad with the mirrored hand, the perceived hardness would be expected to decrease due to decreases in finger displacement of the test hand. However, the perceived hardness was significantly larger when participants pushed the harder pad with the mirrored hand. Therefore, we concluded that modulation of the perceived hardness under the MI-Sync condition is not due to changes in finger movements of the test hand induced by bimanual coordination.

### Effect of Sensory Assimilation

It is well known that the magnitude of stimulus sensation can be influenced by the context in which the stimulus is presented. Assimilation is said to occur when the response to a test stimulus becomes more similar to the response to a distractor presented before or in conjunction with the test stimulus. In the present study, perceived hardness estimates were biased toward the pad touched by the mirrored hand. This modulation was most prominent under the MI-Sync condition, although it was limited and inconsistent under the NI-Sync and RH-Sync conditions. Several studies have revealed that assimilation can occur for somatic sensations. When a test stimulus (either embossed dot patterns or sandpaper) and a distractor of a different roughness are presented to two digits of the same hand, the perceived roughness of the test stimulus is biased toward that of the distractor (Verrillo et al., 1999; Kahrimanovic et al., 2009; Roberts and Humphreys, 2010). Neurons in the somatosensory cortex with a large receptive field covering the neighboring digits (Iwamura et al., 1980; Forss et al., 1995; Biermann et al., 1998) have been thought to play a role in unilateral sensory assimilation (Kahrimanovic et al., 2009; Roberts, 2013).

In contrast, only a few studies have investigated the assimilation of somatic sensations between the two hands. Egeth et al. (1970) reported that the temperature of two metal plates touched by each hand after adaptation to different temperatures is perceived similarly. Roberts (2013) further investigated the assimilation of perceived roughness across hands. When participants compared the roughness of sandpaper touched by each hand sequentially, the perceived roughness of the test stimulus varied with the that of the distractor stimulus presented to the other hand: the test stimulus was perceived as rougher when paired with a rougher distractor, and as smoother when paired with a smoother distractor. These results suggest that assimilation of perceived roughness can occur both within and across hands. Considering that input from slowly adapting type I (SAI) and fast-adapting type I (FAI) mechanoreceptors in the finger pad is important for perceiving both roughness and hardness (Phillips et al., 1992; Condon et al., 2014), such assimilation may also occur across hands when perceiving hardness.

Although we cannot exclude the possibility that the modulatory effect observed under the Sync conditions is attributable in part to the assimilation of tactile input from both hands, we speculate that the effect of assimilation is quite limited for the following reasons. First, if assimilation is the dominant mechanism influencing perceptions of hardness, then modulation of the perceived hardness should be observed consistently under the NI-Sync, RH-Sync and MI-Sync conditions. However, this was not the case in the present study. Modulation was prominent and consistent under the MI-Sync condition for both hands, whereas it was partial and inconsistent under the NI-Sync and RH-Sync conditions. Second, assimilation is expected to occur when two stimuli are presented sequentially, as well as concurrently (Gescheider, 1997; Roberts, 2013). If this were the case, assimilation should have been observed during the Async conditions of the present study, in which participants touched pads of different solidities with both hands in an alternating fashion. However, no modulatory effects were observed during the Async conditions. Thus, we concluded that visual information was the dominant modulatory factor influencing perceived hardness during the MI-Sync condition.

## Data Availability

The raw data supporting the conclusions of this manuscript will be made available by the authors, without undue reservation, to any qualified researcher.

## Author Contributions

NK planned the research project and acquired funding. NK, NU and MT designed the study. NK and EK-T collected the experimental data. HY and AS supported the data collection. NK analyzed the experimental data and prepared the manuscript with support from NU and MT.

## Conflict of Interest Statement

The authors declare that the research was conducted in the absence of any commercial or financial relationships that could be construed as a potential conflict of interest.

## Footnotes

^1^http://www.gpower.hhu.de/
